# A discussion of key values to inform the design and delivery of services for HIV-affected women and couples attempting pregnancy in resource-constrained settings

**DOI:** 10.7448/IAS.18.6.20272

**Published:** 2015-12-01

**Authors:** Renee Heffron, Natasha Davies, Ian Cooke, Angela Kaida, Reid Mergler, Sheryl van der Poel, Craig R Cohen, Okeoma Mmeje

**Affiliations:** 1 Department of Global Health, University of Washington, Seattle, WA, USA;; 2 Department of Epidemiology, University of Washington, Seattle, WA, USA; 3 Wits Reproductive Health and HIV Institute, University of Witwatersrand, Johannesburg, South Africa; 4 Department of Human Metabolism, University of Sheffield, Sheffield, UK; 5 Faculty of Health Sciences, Simon Fraser University, Burnaby, Canada; 6 Albert Einstein College of Medicine, Bronx, NY, USA; 7 Department of Reproductive Health and Research Including the Human Reproduction Research Programme, World Health Organization, Geneva, Switzerland; 8 Department of Obstetrics, Gynecology & Reproductive Sciences, University of California San Francisco, San Francisco, CA, USA; 9 Department of Obstetrics & Gynecology, University of Michigan, Ann Arbor, MI, USA; 10 Department of Health Behavior and Health Education, University of Michigan, Ann Arbor, MI, USA

**Keywords:** HIV, pregnancy, values, reproductive health, fertility, women, couples

## Abstract

**Introduction:**

HIV-affected women and couples often desire children and many accept HIV risk in order to attempt pregnancy and satisfy goals for a family. Risk reduction strategies to mitigate sexual and perinatal HIV transmission include biomedical and behavioural approaches. Current efforts to integrate HIV and reproductive health services offer prime opportunities to incorporate strategies for HIV risk reduction during pregnancy attempts. Key client and provider values about services to optimize pregnancy in the context of HIV risk provide insights for the design and implementation of large-scale “safer conception” programmes.

**Discussion:**

Through our collective experience and discussions at a multi-disciplinary international World Health Organization–convened workshop to initiate the development of guidelines and an algorithm of care to support the delivery of services for HIV-affected women and couples attempting pregnancy, we identified four values that are key to the implementation of these programmes: (1) understanding fertility care and an ability to identify potential fertility problems; (2) providing equity of access to resources enabling informed decision-making about reproductive choices; (3) creating enabling environments that reduce stigma associated with HIV and infertility; and (4) creating enabling environments that encourage disclosure of HIV status and fertility status to partners. Based on these values, recommendations for programmes serving HIV-affected women and couples attempting pregnancy include the following: incorporation of comprehensive reproductive health counselling; training to support the transfer and exchange of knowledge between providers and clients; care environments that reduce the stigma of childbearing among HIV-affected women and couples; support for safe and voluntary disclosure of HIV and fertility status; and increased efforts to engage men in reproductive decision-making at times that align with women's desires.

**Conclusions:**

Programmes, policies and guidelines that integrate HIV treatment and prevention, sexual and reproductive health and fertility care services in a manner responsive to user values and preferences offer opportunities to maximize demand for and use of these services. For HIV-affected women and couples attempting pregnancy, the provision of comprehensive services using available tools – and the development of new tools that are adaptable to many settings and follow consensus recommendations – is a public health imperative. The impetus now is to design and deliver value-driven inclusive programming to achieve the greatest coverage and impact to reduce HIV transmission during pregnancy attempts.

## Introduction

Attempting pregnancy to achieve goals for family size is a basic reproductive right for all individuals, including HIV-affected women and couples, whether they be HIV-infected women with partners of known and unknown HIV status, HIV-serodiscordant couples, or HIV-uninfected women with a high risk for HIV acquisition [1]. HIV-affected women and couples face a dilemma when attempting pregnancy, a period associated with heightened risk of HIV transmission. Many accept increased HIV risk in order to satisfy their fertility goals [2]. Pregnancy rates among HIV-affected women and couples have been documented at 10 to 15% per year, including substantial proportions with partners of unknown or HIV-serodiscordant status and important proportions that are unintended [3]. Antiretroviral use by women is associated with improved health and hopefulness for the future, contributing to desires to have more children while seeking care to minimize HIV risk during pregnancy attempts [4–9]. Thus, there is a great clinical need and a public health imperative to provide services for HIV-affected women and couples desiring pregnancy to maximize the potential for a healthy pregnancy while minimizing sexual and perinatal HIV transmission risks.

Biomedical and behavioural strategies are available to support pregnancy attainment while minimizing HIV risk (i.e. “safer conception” strategies) and international guidelines to optimize pregnancy and HIV outcomes are under development [10]. For HIV-affected women and couples with normal fertility, evidence-based strategies include antiretroviral therapy use by the HIV-infected partner, the use of pre-exposure prophylaxis (PrEP) by the HIV-uninfected partner, limiting condomless sex to periods of peak fertility, treatment of sexually transmitted infections, voluntary medical male circumcision and manual insemination (for serodiscordant couples with an HIV-uninfected male partner) [11–15]. Fertility care services, including fertility screening and management, offer additional options when fertility may be compromised. The availability of multiple strategies offers choice and the possibility of combining strategies to maximize harm reduction.

The integration of HIV and reproductive health care services provides a natural opportunity to incorporate pregnancy planning and fertility management for HIV-affected women and couples attempting pregnancy [16–18]. Models that include comprehensive reproductive, sexual, maternal and child health services – and can accommodate individuals at any stage in their reproductive life – have the potential to ensure more equitable service provision and reduce missed opportunities to prevent HIV transmission, unintended pregnancy and poor pregnancy outcomes [19]. Within this integration, opportunities also arise to create programmes that encourage the engagement of men as supporters of HIV-infected women in reproductive health decision-making and as partners in health optimization prior to and during pregnancy. Thus, recommendations for the design and delivery of services for HIV-affected women and couples attempting pregnancy can be contextualized within the premise of integrated systems, generating programmes that minimize burden, are cost-effective and place HIV prevention and reproductive health care within a holistic and rights-based framework.

This commentary, developed through collaboration by HIV prevention and fertility care experts, discusses key values of HIV-affected women and couples seeking services to reduce HIV risk during pregnancy attempts as well as those of healthcare providers encountering opportunities to initiate discussion about fertility goals and provide pre-pregnancy care. A greater understanding of these values is essential for maximizing the impact of safer conception programmes and services. HIV-affected women and couples can also meet their family goals through adoption, donor gamete or surrogacy, but values related to these methods are beyond the scope of this discussion.

## Discussion

In December 2014, the WHO convened a meeting to initiate the development of guidelines and an algorithm of care to support the delivery of services for HIV-affected women and couples attempting pregnancy. During discussions, the authors identified four key values to incorporate into the development of programmes for HIV-affected women and couples. The attendees were experts in HIV prevention and treatment as well as reproductive medicine whose experiences and knowledge about each other's fields was initially limited. Through structured debate among the attendees, four key values emerged as being central to programmatic recommendations: (1) understanding fertility care and the ability to identify potential fertility problems; (2) providing equity of access to resources enabling informed decision-making about reproductive choices; (3) creating enabling environments that reduce stigma associated with HIV and infertility; and (4) creating enabling environments that encourage disclosure of HIV status and fertility status to partners.

### Understanding fertility care and the ability to identify potential fertility problems

**Figure 1:**
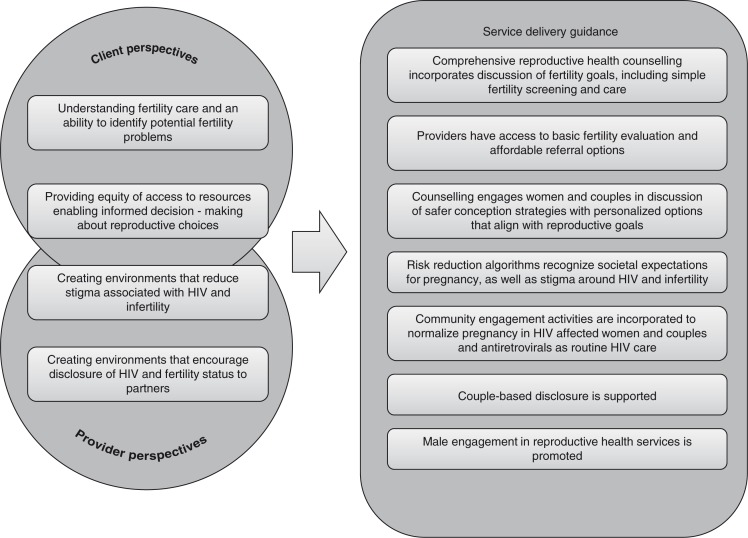
Key values and service delivery guidance for programmes to reduce HIV risk during pregnancy attempts (i.e. safer conception services). The identified key values of HIV-affected women and couples were drawn from client and provider values and preferences and provide guidance for the integration of services to reduce HIV risk during pregnancy attempts. For example, the key value of understanding fertility care and having the ability to identify potential fertility problems is incorporated into guidance that comprehensive reproductive health counselling should incorporate discussion of fertility goals, including simple fertility screening and care. The identification of additional values and service delivery guidance would be warranted through further collaborative discussions.

Fertility awareness or confidence in the ability to become pregnant is often questioned by individuals before pregnancy occurs. HIV-affected women and couples may unknowingly have fertility problems and would value provider counselling on how to evaluate fertility, diagnose infertility and, if needed, receive fertility services before attempting pregnancy in order to minimize condomless sex and thus the potential for HIV transmission. The prevalence of involuntary childlessness due to infertility has been estimated at one in every four couples in developing countries [20]. Thus, providers working with HIV-affected women and couples with fertility aspirations value information that allows: 1) a recommendation to presumed fertile couples to engage in condomless sex timed to peak fertility during the menstrual cycle, or 2) access to basic fertility evaluation and affordable referral options should infertility be suspected (Figure 1).

Simple screening questionnaires that assess reproductive and lifestyle factors have been developed for individuals to self-assess their fertility potential and to facilitate provider-initiated discussions of reproductive desires and likelihood of normal fecundity [8]. Providers can conduct a basic fertility evaluation to understand history of past pregnancy losses, terminations and complications; menstrual cycle abnormalities for the woman; a semen analysis for the man; and assessment of individual and couples’ lifestyle, environmental exposures, STIs and sexual dysfunction that may alter fertility fitness [21]. These provider competencies must be complemented by offering opportunities for HIV-affected women and their partners to ask questions and become knowledgeable about their reproductive potential. Basic fertility interventions, such as vaginal insemination, could be offered in addition to an option of natural conception under the premise of viral suppression with antiretrovirals and/or the use of PrEP. However, if a desired pregnancy has not occurred within 12 months of regular condomless sex without contraception – or after six months when HIV risk is high – then HIV-affected women and couples are likely to be at significant risk of extended HIV exposure with a reduced chance of pregnancy, and affordable advanced diagnostic techniques and care services should be offered.

An infertility diagnosis can be devastating, especially for HIV-affected women and couples who are already struggling with HIV-related stigma. Providers, in anticipation of this additional distress from fertility problems, must be armed with appropriate knowledge, tools and counselling skills to provide basic fertility care, support and referral to infertility services for advanced diagnosis and management as well as HIV prevention options. To respect the reproductive rights of HIV-affected women and couples to achieve fertility goals, HIV and fertility service providers need to collaborate to ensure comprehensive, coordinated care.

### Providing equity of access to resources enabling informed decision-making about reproductive choices

HIV-affected women and couples value autonomy and the ability to make informed decisions about attempting pregnancy in the face of HIV risk and/or undiagnosed fertility problems. Current evidence suggests that women and men have low awareness of HIV risk reduction strategies they can use during pregnancy attempts, despite highly expressed interest in learning about them [2]. Knowledge about accessibility is an important corollary, because PrEP, fertility screening and basic and advanced assisted reproductive interventions are not universally available in resource-limited settings. In addition, women at risk of acquiring HIV who are considering PrEP require complete and balanced safety data about use during pregnancy, including recent data showing that PrEP does not significantly affect the risk of adverse maternal and infant outcomes [22].

Providers require the skills and confidence to provide comprehensive and non-judgmental guidance about suitable HIV risk reduction and fertility care options. Reproductive health and HIV providers must incorporate a broader exploration of reproductive goals into their established counselling on contraceptive uptake, antiretroviral use to prevent perinatal HIV transmission and consistent condom use. Despite general provider empathy with the social and cultural importance of HIV-infected women having children, knowledge and training on how to support women with this goal remains limited [23–25]. Additionally, many providers struggle with the tension between acknowledging the reproductive rights of HIV-affected women and couples and the clinical responsibility to protect partners and future children from HIV transmission and other pregnancy-related consequences [26]. The traditional emphasis of counselling on consistent condom use must shift, acknowledging the tremendous benefit of antiretrovirals (as treatment and PrEP) as HIV prevention strategies that enable safer condomless sex and support couples to attain their pregnancy goals.

Prevailing social and structural barriers may impact HIV-affected women and couples’ ability to utilize HIV and reproductive health services. For example, optimizing pre-pregnancy maternal health is important for pregnancy planning but may be a lower priority for women, particularly in socio-cultural and normative gendered contexts where pressures and expectations to bear children are high [27]. Where women have limited autonomy over reproductive decision-making, the delays involved in testing and treating pre-existing conditions or infections before pregnancy attempts occur may create difficulties for women. Counselling which directly acknowledges and addresses these delays provides opportunities to encourage pre-pregnancy planning and limit disengagement from care. For some women, pregnancy is not a goal. Ensuring their autonomy to decide against pregnancy is also a reproductive right and, therefore, sexual and reproductive health programmes must also support HIV-affected women and couples to make this choice with provision of, or linkage to, comprehensive contraceptive services [1].

### Creating enabling environments that reduce stigma associated with HIV and infertility

HIV-related stigma emerges as a major challenge to HIV-affected women and couples seeking services to minimize HIV risk during pregnancy attempts [28, 29]. To combat social stigma, programmes need to normalize pregnancy and childbearing desires and the use of HIV risk reduction strategies during pregnancy attempts [26]. In addition to HIV-related stigma, HIV-affected women and couples with suspected or diagnosed infertility may suffer psychosocial distress related to childbearing expectations, especially in societies where childbearing remains a strong expectation. This distress may be magnified in a setting where fertility is not routinely assessed and fertility care services that could resolve an infertility problem, and could reduce the stigma, may not be well established or supported [30].

Long-standing community impressions that HIV is associated with shorter life expectancy and potentially discounts a parent from long-term child-rearing may contribute to stigma associated with pregnancy and childbearing among HIV-affected women and couples. For HIV-infected women attempting pregnancy, antiretroviral therapy is a powerful option to reduce HIV transmission risk, with tremendous clinical benefits for the woman and prevention benefits for partners and the future child [13, 31]. However, HIV-infected individuals have long reported barriers to the initiation of antiretrovirals, citing personal and community perceptions that antiretroviral therapy use signals sickness and imminent death [32]. For HIV-affected women and couples, early provider-initiated discussions of fertility goals offer a prime opportunity to normalize antiretroviral initiation within the context of optimizing health prior to pregnancy and maximizing the likelihood of pregnancy [33].

Despite well-established directives on the rights of HIV-affected women and couples to attempt pregnancy, providers have voiced concerns about managing clinical and relationship complexities presented by HIV-serodiscordant couples wishing to become pregnant [26]. Thus, providers would value opportunities to develop skills to counsel non-disclosed HIV-affected women and couples without inadvertently disclosing HIV or subfertility status and triggering a cascade of poor outcomes, including patient/client distrust, couple discord and intimate partner violence [26]. Of utmost importance is that providers are able to counsel about fertility and HIV, with high sensitivity to social and cultural ideas about pregnancy among HIV-affected women and couples and perceptions of infertility.

### Creating enabling environments that encourage disclosure of HIV and fertility status to partners

For HIV-affected women and couples, disclosing HIV status can be one of the strongest links to health optimization, yet the act of disclosure is often met with trepidation and delays are common due to threats of violence, relationship dissolution and rejection [34]. Similarly, disclosure of infertility by HIV-affected women and couples can reduce HIV exposure during pregnancy attempts that are unlikely to succeed, but infertility disclosure may also result in these types of consequences. Environments supportive of reproductive decision-making must aim to foster the attainment of reproductive autonomy and support individual decision-making regarding the disclosure of HIV and/or infertility status to partners and providers. When disclosure is withheld, however, an environment with supportive providers continues working to maximize pre-pregnancy health and address HIV risk reduction.

For HIV-infected women with HIV-uninfected male partners, HIV status disclosure to a male partner potentially increases his personal HIV risk perception, his motivation to employ risk reduction methods and his engagement in reproductive decision-making [35]. When a heterosexual couple is mutually disclosed, male partners can take a more active role supporting reproductive decision-making including planning to delay pregnancy or seek fertility care to attempt pregnancy [26]. Models to increase male engagement and emphasize comfortable spaces for men, as well as women, have been successfully demonstrated in resource-constrained settings and can be adapted for programmes tailored to HIV risk reduction when pregnancy is attempted [36].

## Conclusions

We have identified and described four key values: understanding fertility care and an ability to identify potential fertility problems, providing equity of access to resources enabling informed decision-making about reproductive choices, creating enabling environments that reduce stigma associated with HIV and infertility and creating enabling environments that encourage disclosure of HIV status and fertility status to partners. These key values should remain central to guidelines, policies and programmes being developed to optimize pregnancy outcomes and reduce HIV risk among HIV-affected women and couples during pregnancy attempts. At this unique time in HIV prevention, when the power of antiretrovirals, including PrEP, and fertility interventions to eliminate HIV risk has been well established, HIV-affected women and couples no longer need to accept elevated HIV risk during pregnancy attempts nor forgo their desires for pregnancy. The imminent task for low-resource, high HIV-burdened settings is to develop acceptable and feasible services that are affordable and cost-effective to meet these needs.

Initially in the HIV epidemic, HIV-affected women and couples were discouraged from attempting pregnancy [37, 38]. The field has certainly progressed, as HIV is now a chronic condition in most societies, and there are great opportunities to help HIV-affected women and couples satisfy their fertility goals with minimal HIV risk while strengthening integrated health systems and fostering collaborations between fertility and HIV care providers. HIV-affected women and couples seeking services must encounter confident, informed providers who are equipped to initiate and engage in discussion about fertility desires, fertility care and HIV risk. Service integration provides opportunities for providers from the two disciplines to share experiences and best practices. HIV care providers, for example, can incorporate fertility screening into HIV care, recognize the importance of delaying pregnancy attempts when fertility may be compromised, provide access to fertility care options and provide counsel that recognizes socio-cultural childbearing expectations. In parallel, fertility care providers can adapt their practice to the context of HIV infection and recognize opportunities to reduce HIV risk, counsel about HIV prevention strategies and promote HIV testing and access to care.

Efforts to integrate services to improve outcomes among HIV-affected women and couples attempting pregnancy need to utilize evidence-based tools and approaches consistent with a sexual and reproductive health rights–based context [1]. This includes tools to expand provider knowledge and confidence when providing HIV prevention and fertility services for HIV-affected women and couples, campaigns to increase demand among HIV-affected women and couples for these services and appropriate systems to capture uptake and access to services. Research utilizing implementation science methods to capture usability and feasibility data is needed to evaluate various delivery models and determine the most cost-effective approaches. In addition, international clinical guidelines based on consensus recommendations from HIV prevention and fertility care researchers, providers, advocates and HIV-affected women and couples are urgently needed to spur the development of scalable programmes to reduce HIV risk during attempts to become pregnant.

In this commentary, we have described four key values to prioritize when integrating pre-pregnancy care and counselling services into HIV prevention and reproductive health services for HIV-affected women and couples. We identified many additional values including empowerment, religious beliefs and mental health services, but limited our descriptions here to the four that have dominated our discussions. Importantly, each HIV-affected individual seeking services presents a different HIV risk profile – whether in a seroconcordant partnership with risk of perinatal transmission or superinfection, HIV-uninfected with a partner of unknown status and the risk of sexual and perinatal transmission, unknown subfertility that necessitates prolonged pregnancy attempts or assisted reproductive care, or myriad other scenarios. Individuals prioritize values differently; programmes must consider each of these scenarios to ensure that their services accommodate all.

Programmes responsive to client values and preferences are inherently better positioned to meet client needs, fostering greater demand and uptake. Opportunities to incorporate pregnancy planning and fertility management exist through ongoing efforts to integrate HIV prevention and sexual and reproductive health programmes and the resulting programmes should be poised to fully respond to sexual and reproductive health needs. Minimizing HIV risk during pregnancy attempts for HIV-affected women and couples is a public health imperative. Tools to accomplish safer conception are available – and new tools developed alongside global consensus guidelines will add to this compendium. The impetus now is to develop value-driven, inclusive, scalable programmes to deliver interventions to minimize HIV transmission risk during pregnancy attempts with maximal coverage and impact.
